# Global disparities in the availability and reimbursement of systemic therapies for renal cell carcinoma: results of the International ARON-AID Survey

**DOI:** 10.1093/oncolo/oyag397

**Published:** 2026-10-06

**Authors:** Yuksel Urun, Kannan Sridharan, Anca Zgura, Anas Zayed, Martin Ignacio Zapata Laguado, Tarkan Yetisyigit, Akihiro Yano, Mike Wenzel, Nuno Vau, Deniz Tural, Karine Trindade, Ben Tran, Frederic Ivan Leong Ting, Arnoud J Templeton, Hideki Takeshita, Kaisa Sunela, Vinicius Carrera Souza, Mopa Alina Sooro, Andrey Soares, Shankar Siva, Ashish Singh, Glauco Silveira, Shamik Shah, Emmanuel Seront, Fabio A Schutz, Maitê de Liz Vassen Schurmann, Timothy Schieber, Sarah Scagliarini, Amalya Sargsyan, Carlos Salinas Simonetti, Marine Rushanyan, Giandomenico Roviello, Alejo Rodriguez-Vida, Orion Erenhu Rodriguez Gonzalez, Robin Roberts, Astrid A M Van der Veldt, Mimma Rizzo, Alessandro Rizzo, Alexandr Poprach, Alina Pirshtuk, Alvaro Pinto, Erin Pierce, Jose Mauricio Peñaloza, Martino Pedrani, Se Hoon Park, Ibukun Adewumi Okunade, Karen Flora Odounga, Julio Noriega, Edriss Noorzai, Maria Tereza Nieto Coronel, Jiri Navratil, Zin W Myint, Vladan Mutavdzic, Olivier Mukuku, Robert Mugarura, Fernando Sabino Marques Monteiro, Abdikarim Hussein Mohamed, Daniel Mmereki, Carlo Messina, Bohuslav Melichar, Pitchou Mukaz Mbey, Ernesto Maturana, Diana Matthews, Francesco Massari, Esther Martínez, Priyamvada Maitre, Patrick Maison, Beaud Conrad Mabika Ndjembidouma, Kevin Lu, Jiakun Li, Haoran Li, Luciana de Moura Leite, Aly-Khan A Lalani, Zsófia Küronya, Jakub Kucharz, Arnaud Boris Koumba, Ray Manneh Kopp, Jindrich Kopecky, Alba J Kihn-Alarcón, Özge Keskin, Kymbat Kabdullina, Álvaro Juarez, María José Juan Fita, Denis Leonardo Jardim, Laurianne Grégoria James, Enrique Grande, Alfonso Gómez De Liaño, Patrizia Giannatempo, Marwan Ghosn, Cinthia Gauna, Maria Natalia Gandur Quiroga, Kazutoshi Fujita, Marcelo Roberto Pereira Freitas, Ondřej Fiala, Mauricio Fernandez Lazzaro, Glenda M Falcon Pacheco, Theophilus Olaide Esan, Deborah Tolulope Esan, Danelo Du Plessis, Silvia Neciosup Delgado, Simon J Crabb, Rafael Correa Cano, Luis Corrales, Jixiang Chen, Federico Cayol, Clarissa Cavalin Silva, André Paternò Castello Dias Carneiro, Georgiana Anca Caprinciu, Saul Campos, Thomas Büttner, Sebastiano Buti, Nedal Bukhari, Tomas Buchler, Maria T Bourlon, Hatice Bolek, Martin Boegemann, Mehmet A Bilen, Laura Bernal Vaca, Diogo Assed Bastos, Philippe Barthelemy, Samvel Bardakhchyan, Maria Alexandra Barbu, Jalal Baranseh, Aristotelis Bamias, Alex Bakenga, Georgia Anguera, Martin Angel, Carolina Alves Costa Silva, Teresa Alonso-Gordoa, Fahad Almugbel, Roula Almufti, Thiago Lins Almeida, Altay Aliyev, Carlos Aliaga, Umair Aleem, Erman Akkus, Luis Garcia Aceituno, Syed Hani Abidi, Halima Abahssain, Francesca Tartari, Javier Molina-Cerrillo, Matteo Santoni

**Affiliations:** Department of Medical Oncology, Ankara University Faculty of Medicine, Ankara 06620, Türkiye; Cancer Research Institute, Ankara University, Ankara, Turkiye; Department of Pharmacology & Therapeutics, College of Medicine & Health Sciences, Arabian Gulf University, Kingdom of Bahrain; Department of Obstetrics-Radiotherapy, Alexandru Trestioreanu Institute of Oncology, “Carol Davila” University of Medicine and Pharmacy, Bucharest, Romania; Department of Internal Medicine, King Hussein Cancer Center, Amman, Jordan; Department of Clinical Oncology, Instituto Nacional de Cancerología, Bogotá, Colombia; Department of Medical Oncology, Bahrain Oncology Center, Muharraq, Bahrain; Department of Urology, Saitama Medical Center, Saitama Medical University, Kawagoe, Saitama, Japan; Department of Urology, University Hospital Frankfurt, Goethe University, Frankfurt, Frankfurt am Main, Germany; Urologic Oncology, Champalimaud Clinical Center, Lisbon 1400-038, Portugal; Department of Medical Oncology, Koç University School of Medicine, Istanbul, Turkey; Oncocentro Ceará (Rede D’Or), Fortaleza, Brazil; Department of Medical Oncology, Peter MacCallum Cancer Centre, Melbourne, Australia; Section of Medical Oncology, Department of Internal Medicine, Corazon Locsin Montelibano Memorial Regional Hospital, Bacolod 6100, Philippines; Department of Clinical Sciences, College of Medicine, University of St. La Salle, Bacolod 6100, Philippines; Faculty of Medicine, University of Basel, Basel, Switzerland; Department of Oncology, St. Claraspital, Basel, Switzerland; St. Clara Research, Basel, Switzerland; International University of Health and Welfare Hospital, 852 Hatakeda, Narita-shi, Chiba 286-8520, Japan; Finnish Medicines Agency, Tampere, Finland; Oncologia D’OR, Salvador, Brazil; Jiangsu Key Laboratory of New Drug Screening & Jiangsu Center for Pharmacodynamics Research and Evaluation, China Pharmaceutical University, Nanjing 210009, China; Latin American Cooperative Oncology Group—LACOG, Porto Alegre, Brazil; Hospital Israelita Albert Einstein, São Paulo, SP, Brazil; Department of Radiation Oncology and Sir Peter MacCallum Department of Oncology, Peter MacCallum Cancer Centre, University of Melbourne, Victoria, Australia; Department of Medical Oncology, Christian Medical College, Vellore, Tamil Nadu 632004, India; Centro Oncológico do Triângulo, Uberlândia, Brazil; Department of Nephrology, King Hamad American Mission Hospital, Kingdom of Bahrain; Department of Medical Oncology, Cliniques Universitaires Saint Luc, Brussel, Belgium; Latin American Cooperative Oncology Group—LACOG, Porto Alegre, Brazil; Beneficência Portuguesa de São Paulo, São Paulo, SP, Brazil; ANIMI—Unidad de Tratamento Oncológico, Lages, SC, Brazil; Division of Medical Oncology, Department of Internal Medicine, University of Kansas Cancer Center, Westwood, KS, United States; Department of Medical Oncology, AORN “A. Cardarelli,”Naples, Italy; Immune Oncology Research Institute, 7 Nersisyan, Yerevan 0014, Armenia; Yeolyan Hematology and Oncology Center, Yerevan, Armenia; Yerevan State Medical University, Yerevan, Armenia; Medical Oncology Service, Bradford Hill, Santiago de Chile, Chile; Yeolyan Hematology and Oncology Center, Yerevan, Armenia; Department of Oncology, Gyumri Medical Center, Gyumri, Armenia; Department of Health Sciences, University of Florence, Florence, Italy; Hospital del Mar Research Institute, Barcelona, Spain; Escuela Militar de Graduados de Sanidad, Batalla de Celaya 202, Lomas de Sotelo, Militar, Miguel Hidalgo, Ciudad de México 11200, Mexico; Hospital Militar Regional de Especialidades Guadalajara, Reforma, Colonia Centro, C.P. 44890, Guadalajara, Jalisco, Mexico; UWI School of Clinical Medicine and Research, Nassau, The Bahamas; Department of Medical Oncology, Erasmus MC Cancer Institute, Rotterdam, The Netherlands; Medical Oncology Unit, Azienda Ospedaliera Universitaria Consorziale Policlinico di Bari, Bari, Italy; S.S.D. C.O.r.O. Bed Management Presa in Carico, TDM, IRCCS Istituto Tumori “Giovanni Paolo II,”Bari, Italy; Masaryk Memorial Cancer Institute, Brno, Czech Republic; Faculty of Medicine, Masaryk University, Brno, Czech Republic; Department of Oncology, Second Faculty of Medicine, Charles University and University Hospital Motol, Prague, Czech Republic; Department of Medical Oncology, Hospital Universitario La Paz, Madrid, Spain; HonorHealth Research Institute, 10510 N. 92nd St., Suite 100, Scottsdale, AZ 85258, United States; Centro Oncológico Integral, Neuquén, Argentina; Istituto Oncologico Svizzera Italiana, Ente Ospedaliero Cantonale, Ospedale Regionale Bellinzona e Valli, Bellinzona 6500, Switzerland; Samsung Medical Center, Sungkyunkwan University School of Medicine, Seoul, South Korea; Bowen University Teaching Hospital, P.O. Box 15, Ogbomoso, Oyo State, Nigeria; Bowen University, P.M.B. 284, Iwo, Osun State, Nigeria; Service Pharmacie, Institut de Cancérologie de Libreville, Libreville, Gabon; Fox Chase Cancer Center, Philadelphia, PA 19111, United States; Peninsula Health, Hastings Road, Frankston, VIC 3199, Australia; Oncopalia.MyA Center, Medical Oncology Departament, La Paz City, Bolivia; Masaryk Memorial Cancer Institute, Brno, Czech Republic; Faculty of Medicine, Masaryk University, Brno, Czech Republic; Division of Medical Oncology, Markey Cancer Center, University of Kentucky, Lexington, KY, United States; Department of Oncology, Faculty of Medical Sciences, University of Kragujevac, Kragujevac 34000, Serbia; University Clinical Center Kragujevac, Kragujevac 34000, Serbia; Department of Maternal and Child Health, High Institute of Medical Techniques, Lubumbashi, Democratic Republic of the Congo; Department of Surgery, Kabale University, Kabale District, Uganda; Latin American Cooperative Oncology Group—LACOG, Porto Alegre, Brazil; ARON Research Foundation ETS, Galleria del Commercio n. 6, Macerata, Italy; Hospital Sírio-Libanês, Brasília, DF, Brazil; Kaafi Hospital, Mogadishu, Somalia; Faculty of Health Sciences, Division of Radiation Oncology, University of the Witwatersrand, Johannesburg 2193, South Africa; Oncology Unit, UOC Oncologia ARNAS Civico Benfratelli di Cristina, Piazza Nicola Leotta 4, Palermo 90127, Italy; Faculty of Medicine and Dentistry, Department of Oncology, Palacký University Olomouc and University Hospital, Zdravotníků 248, Olomouc 775 20, Czech Republic; Department of Surgery, Faculty of Medicine, University of Lubumbashi, Lubumbashi, Democratic Republic of the Congo; Department of Surgery, Urology, Faculty of Medicine, University of Kinshasa, Kinshasa, Democratic Republic of the Congo; Pontificia Universidad Católica de Chile, Avenida Libertador Bernardo O’Higgins 340, Santiago, CL-8320000, Chile; Velindre Cancer Centre, Velindre Road, Whitchurch, Cardiff, Wales; ARON Research Foundation ETS, Galleria del Commercio n. 6, Macerata, Italy; Medical Oncology, IRCCS Azienda Ospedaliero-Universitaria di Bologna, Bologna, Italy; Department of Medical and Surgical Sciences (DIMEC), University of Bologna, Bologna, Italy; Hospital Universitario de Jaén, Jaén, Spain; Radiation Oncology, Advanced Centre for Treatment, Research and Education in Cancer (ACTREC), and Tata Memorial Hospital (TMH), Homi Bhabha National Institute, Mumbai 400094, India; Department of Surgery, School of Medical Sciences, University of Cape Coast, Cape Coast, Ghana; Departement of Medical Physics and Radiotherapy, Akanda Cancer Institute, Libreville, Gabon; Department of Urology, Kaohsiung Medical University Hospital & College of Medicine, Kaohsiung Medical University; Department of Urology, West China Hospital, Sichuan University, Chengdu, Sichuan, PR China; Division of Medical Oncology, Department of Internal Medicine, University of Kansas Cancer Center, Westwood, KS, United States; AC Camargo Cancer Center; Department of Medical Oncology, Juravinski Cancer Centre, McMaster University, Hamilton, Canada; Urogenitális Tumorok és Klinikai Farmakológiai Osztály, Országos Onkológiai Intézet, Budapest, Hungary; Department of Clinical Oncology, Semmelweis University; Department of Uro-Oncology, Maria Sklodowska-Curie National Research Institute of Oncology, Warsaw, Poland; Department of Nuclear Medicine, Akanda Cancer Institute, Libreville, Gabon; Clinical Oncology, Sociedad de Oncología y Hematología del Cesar, Valledupar, Colombia; Department of Clinical Oncology, and Radiotherapy, University Hospital Hradec Kralove, Hradec Kralove, Czech Republic; Research Department, Liga Nacional Contra el Cáncer & Instituto de Cancerología, 6a Avenida 6-58, Cdad, de Guatemala 01011, Guatemala; Medical Oncology Department, Bahrain Oncology Center, King Hamad University Hospital, AlMuharraq, Bahrain; Department of Biomedical Sciences, Nazarbayev University School of Medicine, Astana, Kazakhstan; Department of Urology, Jerez University Hospital, Jerez, Spain; Department of Medical Oncology, Fundación Instituto Valenciano de Oncología (IVO), Valencia, Spain; Oncology Center, Hospital Sírio-Libanês, São Paulo, Brazil; Grupo Oncoclínicas, São Paulo, SP, Brazil; Departement of Medical Physics and Radiotherapy, Akanda Cancer Institute, Libreville, Gabon; Department of Medical Oncology, Quirónsalud Madrid, Spain; Medical Oncology Department, CHU Insular-Materno Infantil, Las Palmas, Spain; Dipartimento di Oncologia Medica, Fondazione IRCCS Istituto Nazionale dei Tumori, Milan, Italy; Department of Hematology-Oncology, Hotel-Dieu de France University Hospital, Beirut, Lebanon; Instituto Nacional del Cáncer, Capiatá, Paraguay; Division of Genitourinary Medical Oncology, Instituto de Oncologia Ángel H. Roffo, Buenos Aires, Argentina; Department of Urology, Faculty of Medicine, Kindai University, Osaka, Japan; Centro de Pesquisas Oncológicas—CEPON, Florianópolis, SC, Brazil; ARON Research Foundation ETS, Galleria del Commercio n. 6, Macerata, Italy; Department of Oncology and Radiotherapeutics, Faculty of Medicine and University Hospital in Pilsen, Charles University, Pilsen, Czech Republic; Biomedical Center, Faculty of Medicine in Pilsen, Charles University, Pilsen, Czech Republic; Department of Uro-Oncology, Fundacion COIR, Mendoza M5500, Argentina; Instituto Regional de Enfermedades Neoplásicas del Sur, Arequipa, Perú; Federal Teaching Hospital, Ido-Ekiti, Nigeria; Faculty of Nursing Sciences, College of Health Sciences, Bowen University, Iwo, Nigeria; Division of Urology, Faculty of Health Science, University of Stellenbosch, Cape Town 7505, South Africa; Tygerberg Hospital, Francie van Zijl Avenue, Parow, Cape Town, Western Cape 7505, South Africa; Department of Medical Oncology, National Institute of Neoplastic Diseases, Lima, Peru; Cancer Research UK Southampton Clinical Trials Unit, University of Southampton and University Hospital Southampton NHS Foundation Trust, Southampton, Hampshire, United Kingdom; Department of Geriatric Oncology, Instituto Nacional de Ciencias Médicas y Nutrición “Salvador Zubirán,”Ciudad de México, México; Department of Medical Oncology, Center for Cancer Research and Management (CIMCA), San Jose, Costa Rica; Department of Urology, West China Hospital, Sichuan University, Chengdu, Sichuan, PR China; Sección de Oncología, Servicio de Medicina Interna, Hospital Italiano de Buenos Aires, Buenos Aires, Argentina; Grupo Oncoclínicas, São Paulo, SP, Brazil; CardioHub Medicina Diagnóstica, Rio de Janeiro, Brazil; Latin American Cooperative Oncology Group—LACOG, Porto Alegre, Brazil; Hospital Israelita Albert Einstein, São Paulo, SP, Brazil; Hospital Municipal Vila Santa Catarina, São Paulo, SP, Brazil; Oncologie (Clinica), Affidea—Sema Parc Bucuresti, Romania; Latin American Cooperative Oncology Group—LACOG, Porto Alegre, Brazil; Centro Oncologico Estatal “Dr José Luis Barrera Franco” del ISSEMYM, Toluca de Lerdo, Mexico; Department of Urology, and Paediatric Urology, University Hospital Bonn, Bonn, Germany; ARON Research Foundation ETS, Galleria del Commercio n. 6, Macerata, Italy; Medical Oncology Unit, University Hospital of Parma, Parma, Italy; Department of Medicine and Surgery, University of Parma, Parma, Italy; Oncology Department, Prince Sultan Military Medical City, Riyadh, Saudi Arabia; Department of Oncology, Second Faculty of Medicine, Charles University and University Hospital Motol, Prague, Czech Republic; Hematology and Oncology Department, Instituto Nacional de Ciencias Médicas y Nutrición Salvador Zubirán, Mexico City, Mexico; Department of Medical Oncology, Ankara University, School of Medicine, Ankara 06590, Turkey; Ankara University Cancer Institute, Ankara, Turkey; Department of Urology, University of Muenster Medical Center, Muenster, Germany; West German Cancer Center, Muenster, Germany; Department of Hematology and Medical Oncology, Winship Cancer Institute, Emory University, Atlanta, GA, United States; Servicio de Oncología, Clínica Universitaria Colombia—Clínica de Marly, Bogotá, Colombia; Hospital Sirio Libanes, Sao Paulo, Sao Paulo, Brazil; Department of Medical Oncology, ICANS, Strasbourg, France; Immune Oncology Research Institute, 7 Nersisyan, Yerevan 0014, Armenia; Yeolyan Hematology and Oncology Center, Yerevan, Armenia; Department 8-Radiology, Oncology, Hematology, Faculty of Medicine, “Carol Davila” University of Medicine, and Pharmacy Bucharest Romania; MedEuropa Center of Oncology and Radiation Therapy Bucharest Romania; Oncology Wing, RAMBAM Health Care Campus, Haifa, Israel; 2nd Propaedeutic Department of Internal Medicine, ATTIKON University Hospital, School of Medicine, National and Kapodistrian University of Athens, Athens, Greece; Department of Medical Oncology, and Hematology, Uganda Cancer Institute, Kampala, Uganda; Department of Medical Oncology, Hospital de La Santa Creu i Sant Pau, Barcelona, Spain; Clinical Oncology, Genitourinary Oncology Unit, Alexander Fleming Institute, Buenos Aires, Argentina; Gonçalo Moniz Institute, Fiocruz, Salvador, Bahia, Brazil; Medical Oncology Department, School of Medicine, Federal University of Bahia, Salvador, Bahia, Brazil; Medical Oncology Department, Hospital Universitario Ramón y Cajal, Madrid, Spain; Cancer Center of Excellence, King Faisal Specialist Hospital & Research Centre, Riyadh 11211, Saudi Arabia; Medical Oncology Department, King Faisal Specialist Hospital and Research Centre, Jeddah, Saudi Arabia; Grupo Oncoclínicas, São Paulo, SP, Brazil; Medical Oncology Department, Universidade Federal da Paraíba, Cidade Universitária, Brazil; Department of Oncology, Liv Bona Dea Hospital, Baku, Azerbaijan; Servicio de Oncología, Centro Oncológico Aliada, Lima 15036, Peru; Department of Medical Oncology, The Christie NHS Foundation Trust, 550 Wilmslow Road, Withington, Manchester, M20 4BX, United Kingdom; Department of Medical Oncology, Ankara University Faculty of Medicine, Ankara 06620, Türkiye; Cancer Research Institute, Ankara University, Ankara, Turkiye; Latin American Cooperative Oncology Group—LACOG, Porto Alegre, Brazil; Clinica Medica Especializada en Oncologia Medica, Guatemala City, Guatemala; Department of Biomedical Sciences, Nazarbayev University School of Medicine, Astana, Kazakhstan; Oncology and Medical Specialties Department, Valenciennes General Hospital, Valenciennes 59300, France; Equipe de Recherche en Oncologie Translationnelle (EROT), Faculty of Medicine and Pharmacy, University Mohammed V in Rabat, Rabat 10100, Morocco; ARON Research Foundation ETS, Galleria del Commercio n. 6, Macerata, Italy; ARON Research Foundation ETS, Galleria del Commercio n. 6, Macerata, Italy; Medical Oncology Department, Hospital Universitario Ramón y Cajal, Madrid, Spain; ARON Research Foundation ETS, Galleria del Commercio n. 6, Macerata, Italy; Medical Oncology Unit, Macerata Hospital, Macerata, Italy

**Keywords:** access to cures, ARON, drug availability, economic burden, real-world data, renal cell carcinoma

## Abstract

**Background:**

Renal Cell Carcinoma (RCC) represents a growing global health burden, with rising incidence and mortality worldwide. Despite major therapeutic advances, access to novel systemic treatments remains uneven across countries and regions.

**Methods:**

We conducted a cross-sectional, web-based international survey to assess the availability and reimbursement status of 20 innovative systemic therapies for RCC. Responses were collected from 144 clinicians, including medical oncologists, radiation oncologists, urologists, and nuclear medicine specialists, across 58 countries. Analyses examined associations between national income level, gross domestic product (GDP), and treatment availability.

**Findings:**

Thirty-six percent of the countries analyzed had at least 15 of the 20 therapies available. Full reimbursement for at least 15 of the selected innovative therapies was reported in six countries (10%), while 21 countries (36%) fully reimbursed 10-14 therapies and 13 countries (23%) reimbursed 1-9 therapies. In contrast, no full reimbursement for any of the assessed therapies was reported in 18 countries (31%). Higher national income level was significantly associated with greater availability of innovative therapies (Kruskal–Wallis test, *P* < .0001). Armenia (0.709), Greece (0.685), and Israel (0.666) exhibited the highest GDP-adjusted Drug Availability Index (DAI), whereas India (5.195), Nigeria (4.612), and Morocco (3.794) demonstrated the greatest number of available therapies per US$1000 of GDP per capita.

**Interpretation:**

Access to innovative systemic therapies for RCC remains highly unequal at the global level, with reimbursement posing a major barrier even where drugs are available. Internationally coordinated efforts addressing health policy, infrastructure, and equitable pricing are urgently required to ensure that advances in RCC treatment translate into improved outcomes for patients across diverse economic settings.

Implications for practice Global access to systemic therapies for renal cell carcinoma remains highly heterogeneous, with substantial differences in both drug availability and reimbursement across countries. These disparities may directly influence treatment selection and limit the implementation of guideline-recommended strategies, particularly in resource-constrained settings. Clinicians should therefore consider the local availability and reimbursement landscape when developing treatment strategies and, where preferred regimens are inaccessible, use evidence-based alternatives appropriate to the clinical and economic context. At a health-system level, these findings support efforts to accelerate regulatory and reimbursement pathways, promote equitable pricing and pooled procurement, and expand clinical-trial and access programs to reduce avoidable disparities in RCC care.

## Introduction

Kidney cancer represents a significant global health burden, with an estimated annual incidence of approximately 400 000 new cases and around 175 000 deaths worldwide.^1^ Several risk factors have been identified for kidney cancer, including obesity, hypertension, smoking, and occupational exposure to toxic substances.^2^ Among the various types of kidney cancer, renal cell carcinoma (RCC) accounts for approximately 2% of all cancers and cancer-related mortality worldwide and is the ninth most common cancer type in the United States.^3^ RCC is frequently diagnosed incidentally; however, once metastatic, the 5-year survival rate decreases dramatically to approximately 12%.^3^ More recent real-world registry data suggest modest improvement in outcomes following the introduction of targeted and immunotherapies.^4^

Marked geographic disparities in RCC incidence have been reported. Incidence rates among men are highest in Northern and Eastern Europe and among Black populations in the United States, exceeding 15 cases per 100 000, whereas rates as low as 1 per 100 000 have been observed in some African countries.^5^ Although a substantial increase in new cases has been reported in certain regions, such as Latin America, significant improvements in mortality have been observed in Western and Northern Europe, the United States, and Australia. In contrast, unfavorable mortality trends persist among men in Southern Europe and in low- and middle-income countries, where access to effective treatment options remains limited.^5^

More than 90% of RCC cases fall into one of three main histological subtypes: clear cell, papillary, or chromophobe.^6^ A large global study involving approximately 2000 RCC patients across 43 countries demonstrated considerable variability in patient knowledge and understanding of RCC. Nearly one-third of participants were unaware of treatment selection options for their specific subtype, although 90% expressed willingness to participate in clinical trials.^7^

Over the past two decades, the therapeutic landscape of RCC has undergone a dramatic transformation, transitioning from cytokine-based therapies with limited efficacy to targeted agents and immune checkpoint inhibitors that have substantially improved patient outcomes. VEGFR-targeted tyrosine kinase inhibitors, such as sunitinib, represented the first major therapeutic advance, improving progression-free and overall survival compared with interferon-based therapy.^8^ Immune checkpoint inhibitors subsequently transformed the treatment of advanced RCC, used either alone or in combination with TKIs, and combination immunotherapy is now widely adopted as the preferred frontline approach in clinical practice guidelines.^9^

Treatment strategies for RCC largely depend on disease stage. For localized RCC, recommended nonsurgical options include radiofrequency ablation, stereotactic body radiotherapy, microwave ablation, and cryoablation, while partial nephrectomy is preferred for tumors smaller than 7 cm.^9^ In advanced or metastatic RCC, several systemic therapies are available worldwide, although the specific agents accessible to any given patient vary considerably by country of practice, including tyrosine kinase inhibitors (lenvatinib, axitinib, cabozantinib, sunitinib, pazopanib, sorafenib, and tivozanib), immunotherapies (pembrolizumab, nivolumab, avelumab, ipilimumab, and toripalimab), and other agents such as everolimus (a mammalian target of rapamycin inhibitor) and bevacizumab (a monoclonal antibody targeting vascular endothelial growth factor), and belzutifan, a first-in-class hypoxia-inducible factor 2-alpha (HIF-2α) inhibitor approved for previously treated advanced RCC and for RCC associated with von Hippel–Lindau disease, either as monotherapies or in combination regimens.^10^

Despite established guidelines recommending combination immunotherapy as first-line treatment for metastatic RCC, a recent cohort study of 8534 patients revealed that these regimens are infrequently used in clinical practice. Additionally, substantial patient attrition was observed in later stages of the disease, highlighting a critical gap between guideline recommendations and real-world practice and underscoring the need for improved implementation strategies.^11^ The high acquisition cost of many of these novel agents, often several thousand US dollars per patient per month, represents an additional barrier to their incorporation into national formularies, particularly in resource-limited settings, and may account for part of the disparities in access described below.^12^ One of the major challenges limiting the use of systemic therapies remains their availability and affordability. In this study, the term “access to cures” is used broadly to denote the availability and reimbursement of guideline-recommended systemic therapies with demonstrated efficacy in prolonging survival or achieving durable disease control in RCC, rather than access to a curative treatment in the strict sense. In light of the limited literature on this topic, we conducted the present global survey, promoted by the ARON Research Foundation ETS, involving uro-oncologists, radiotherapists, and nuclear medicine physicians aimed to explore the global drug availability and access to cures for RCC patients, both in the advanced disease setting and as adjuvant therapy.

## Methods

An international, cross-sectional, web-based survey was undertaken to assess the availability and reimbursement of systemic therapies for RCC across countries representing diverse healthcare systems and economic settings.

The primary aim of the study was to evaluate the global availability of guideline-recommended systemic treatments for both localized RCC and advanced RCC (mRCC). The secondary aim was to comprehensively describe between-country variability in reimbursement mechanisms for these systemic therapies.

The survey tool was designed by a multidisciplinary steering committee consisting of medical oncologists, urologists, and radiation oncologists with expertise in RCC management. Questionnaire content was informed by current international treatment guidelines and prior global studies examining the availability, accessibility, and patient-related costs of antineoplastic agents.

Participants were identified through the network of ARON Research Foundation collaborators and affiliated genitourinary oncology cooperative groups and were invited by email to complete the survey on a voluntary basis; no formal random sampling frame was used. This convenience-sampling approach may introduce selection bias, as noted in the Limitations section.

The questionnaire was developed in English and underwent multiple rounds of revision by the steering committee to ensure clarity, content validity, and feasibility. A pilot version was subsequently reviewed by five physicians (M.R., L.C., F.S.M.M., O.F., and M.S.) from different geographic regions to assess comprehensibility, estimate completion time, and collect suggestions for refinement. All proposed modifications were reviewed and approved by the steering committee.

The survey was administered via an online platform created using Elementor (version 3.25.4) and was open to respondents from January 31, 2025 to May 1, 2025. Participation was voluntary, and no financial or other incentives were offered.

The final questionnaire consisted of 40 closed-ended questions (provided in the Supplementary Material) organized into three sections: (1) respondent characteristics, including country of practice and medical specialty (oncology, urology, or radiation oncology); (2) systemic therapies for localized disease, focusing on drug availability and reimbursement in the adjuvant setting; and (3) systemic therapies for advanced or metastatic disease, addressing the availability and reimbursement of immune checkpoint inhibitors and targeted agents, administered as monotherapy or in combination.

Respondents were asked to report on the accessibility of innovative systemic therapies recommended by current guidelines for the treatment of both localized RCC and mRCC in their country of practice, as well as the applicable reimbursement frameworks. For each drug or drug combination approved for a given clinical indication, participants answered two sequential questions: first, whether the treatment was available in their country (available vs not available), and second, the reimbursement status. Reimbursement options were categorized as coverage exclusively through private insurance, full reimbursement by the public healthcare system, or partial public reimbursement.

Countries were grouped by geographic region and income level according to the World Bank classification system.^13^ We additionally examined the association between countries’ 2024 Gross Domestic Product (GDP), as reported on the World Bank website,^13^ and the availability of drugs or therapeutic combinations for RCC patients. The GDP per capita was calculated through the estimated 2024 population data from the World Bank website^13^ for each country. To account for economic differences, we calculated the GDP-adjusted Drug Availability Index (DAI) and the “Drugs per GDP” metric, defined as the number of available drugs per 1000 USD of GDP per capita, using the following formulas:

Both indices were developed for this analysis to complement one another. The GDP-adjusted DAI uses a natural-log transformation of GDP, which compresses the scale of national income and is therefore well suited to describe a general, nonlinear association between economic capacity and drug availability across the full income range; however, this transformation attenuates differences among lower- and middle-income countries with comparatively small absolute GDP differences. The drugs-per-GDP metric, by dividing the number of available drugs directly by GDP per capita, provides a linear measure of the availability achieved relative to a country’s economic capacity, allowing identification of countries that achieve comparatively high therapeutic access relative to national income. This approach is conceptually related to previous affordability and access indices used in global oncology drug-access research,^14^^,^^15^ although the specific formulation applied here is original to this study and has not been previously validated; results based on this index should therefore be interpreted as hypothesis-generating:


$$\mathrm{GDP}-\mathrm{adjusted}\;{\mathrm{DAI}}_{i}=\frac{{\mathrm{Drugs}}_{i}}{\ln({\mathrm{GDP}}_{i})},$$

$$\mathrm{Drugs}\;\mathrm{per}\;{\mathrm{GDP}}_{i}=\frac{{\mathrm{Drugs}}_{i}}{\mathrm{GDP}\;\mathrm{per}\;{{\mathrm{capita}}_{i}\;(\mathrm{USD})}_{\;}}\times 1000,$$

where more than one respondent from the same country completed the survey, responses were compared for consistency across respondents (discrepancies were resolved by the majority of responses). Multiple respondents per country were sought because reimbursement can vary by institution type, region, or insurance scheme within a country, and a single respondent’s institutional experience may not generalize to national policy.

### Ethical considerations

This study was a noninterventional, anonymous survey conducted among healthcare professionals and did not involve patient data or influence clinical decision-making. In accordance with applicable local regulations, approval from an institutional review board or ethics committee was not required. Completion of the questionnaire was considered to constitute informed consent to participate.

## Results

### Participant characteristics

A total of 144 clinicians took part in the survey, comprising 120 medical oncologists (83%), 19 urologists (13%), four radiation oncologists (3%), and one nuclear medicine physician (1%) (Supplementary Figure S1). These specialists are affiliated with institutions across multiple global regions: North America (six clinicians, 4%), Latin America (39 clinicians, 27%), Europe (56 clinicians, 39%), Asia (29 clinicians, 20%), and Africa (14 clinicians, 10%).

The 58 countries represented by the participants and their World Bank income classifications^13^ are shown in Supplementary Figure S2. Information on estimated populations, 2024 GDP and calculated per capita GDP of each participating country are reported in Table 1. In terms of economic grouping, low-income countries accounted for 6% of participants, lower-middle income countries for 28%, upper-middle income countries for 25%, and high-income countries for 41% (Supplementary Figure S2).

**Table 1.** Regional classification, income groups,^16^ estimated 2024 population GDP and GDP per capita.

Country name	Code	Region	2024 GDP (US$)	Estimated population (2024)	2024 GDP per capita (US$)	Income group
**Afghanistan**	AFG	Middle East, North Africa, Afghanistan, & Pakistan	N.A.	42 647 492	N.A.	Low income
**Albania**	ALB	Europe & Central Asia	27 046 429 297	2 377 128	1 137 778	Upper middle income
**Argentina**	ARG	Latin America & Caribbean	638 365 455 340	45 696 159	1 396 978	Upper middle income
**Armenia**	ARM	Europe & Central Asia	25 955 275 380	3 033 500	855 621	Upper middle income
**Australia**	AUS	East Asia & Pacific	1 757 022 451 653	27 196 812	6 460 399	High income
**Azerbaijan**	AZE	Europe & Central Asia	74 315 882 353	10 202 830	728 385	Upper middle income
**Bahamas, The**	BHS	Latin America & Caribbean	15 832 800 000	401 283	3 945 545	High income
**Bahrain**	BHR	Middle East, North Africa, Afghanistan, & Pakistan	47 109 734 309	1 588 670	2 965 357	High income
**Belgium**	BEL	Europe & Central Asia	671 370 081 636	11 858 610	5 661 457	High income
**Bolivia**	BOL	Latin America & Caribbean	54 881 327 453	12 413 315	442 117	Lower middle income
**Brazil**	BRA	Latin America & Caribbean	2 185 821 648 944	211 998 573	1 031 055	Upper middle income
**Canada**	CAN	North America	2 243 636 826 634	41 288 599	5 434 035	High income
**Chile**	CHL	Latin America & Caribbean	330 267 137 372	19 764 771	1 670 989	High income
**China**	CHN	East Asia & Pacific	18 743 803 170 827	1 408 975 000	1 330 315	Upper middle income
**Colombia**	COL	Latin America & Caribbean	418 818 154 879	52 886 363	791 921	Upper middle income
**Congo, Dem. Rep.**	COD	Sub-Saharan Africa	70 962 185 791	109 276 265	64 938	Low income
**Costa Rica**	CRI	Latin America & Caribbean	95 350 423 177	5 129 910	1 858 715	High income
**Czechia**	CZE	Europe & Central Asia	347 034 062 928	10 905 028	3 182 331	High income
**Finland**	FIN	Europe & Central Asia	298 696 961 298	5 619 911	5 314 977	High income
**France**	FRA	Europe & Central Asia	3 160 442 622 465	68 551 653	4 610 308	High income
**Gabon**	GAB	Sub-Saharan Africa	20 895 684 426	2 538 952	823 004	Upper middle income
**Germany**	DEU	Europe & Central Asia	4 685 592 577 805	83 516 593	5 610 373	High income
**Ghana**	GHA	Sub-Saharan Africa	82 308 110 386	34 427 414	239 077	Lower middle income
**Greece**	GRC	Europe & Central Asia	256 238 371 778	10 405 134	2 462 615	High income
**Guatemala**	GTM	Latin America & Caribbean	113 199 581 158	18 406 359	615 003	Upper middle income
**Hungary**	HUN	Europe & Central Asia	222 722 738 926	9 562 065	2 329 233	High income
**India**	IND	South Asia	3 909 891 533 858	1 450 935 791	269 474	Lower middle income
**Indonesia**	IDN	East Asia & Pacific	1 396 300 098 191	283 487 931	492 543	Upper middle income
**Ireland**	IRL	Europe & Central Asia	609 157 459 747	5 395 790	11 289 495	High income
**Israel**	ISR	Middle East, North Africa, Afghanistan, & Pakistan	540 379 921 262	9 974 400	5 417 668	High income
**Italy**	ITA	Europe & Central Asia	2 380 825 077 244	58 952 704	4 038 534	High income
**Japan**	JPN	East Asia & Pacific	4 027 597 523 551	123 975 371	3 248 708	High income
**Jordan**	JOR	Middle East, North Africa, Afghanistan, & Pakistan	53 352 289 577	11 552 876	461 810	Lower middle income
**Kazakhstan**	KAZ	Europe & Central Asia	291 480 274 649	20 592 571	1 415 463	Upper middle income
**Lebanon**	LBN	Middle East, North Africa, Afghanistan, & Pakistan	N.A.	N.A.	N.A.	Lower middle income
**Lesotho**	LSO	Sub-Saharan Africa	2 271 759 455	2 337 423	97 191	Lower middle income
**Mexico**	MAR	Middle East, North Africa, Afghanistan, & Pakistan	1 856 365 616 166	130 861 007	1 418 578	Lower middle income
**Morocco**	MEX	Latin America & Caribbean	160 610 994 055	38 081 173	421 760	Upper middle income
**Netherlands**	NLD	Europe & Central Asia	1 214 927 698 573	17 993 485	6 752 042	High income
**Nigeria**	NGA	Sub-Saharan Africa	252 261 880 141	232 679 478	108 416	Lower middle income
**Paraguay**	PRY	Latin America & Caribbean	44 458 118 397	6 929 153	641 610	Upper middle income
**Peru**	PER	Latin America & Caribbean	289 221 969 063	34 217 848	845 237	Upper middle income
**Philippines**	PHL	East Asia & Pacific	461 617 509 782	115 843 670	398 483	Lower middle income
**Poland**	POL	Europe & Central Asia	917 767 106 147	36 559 233	2 510 357	High income
**Portugal**	PRT	Europe & Central Asia	313 271 185 085	10 694 681	2 929 224	High income
**Romania**	ROU	Europe & Central Asia	382 564 217 989	19 051 804	2 008 021	High income
**Saudi Arabia**	SAU	Middle East, North Africa, Afghanistan, & Pakistan	1 239 804 533 333	35 300 280	3 512 166	High income
**Serbia**	SRB	Europe & Central Asia	90 097 765 959	6 586 476	1 367 921	Upper middle income
**Somalia, Fed. Rep.**	SOM	Sub-Saharan Africa	11 967 000 000	19 009 151	62 954	Low income
**South Africa**	ZAF	Sub-Saharan Africa	401 144 998 374	64 007 187	626 719	Upper middle income
**South Korea**	KOR	East Asia & Pacific	1 875 388 209 407	51 751 065	3 623 864	High income
**Spain**	ESP	Europe & Central Asia	1 725 671 652 742	48 848 840	3 532 677	High income
**Switzerland**	CHE	Europe & Central Asia	936 564 198 049	9 005 582	10 399 819	High income
**Taiwan**	TWN	East Asia & Pacific	N.A.	N.A.	N.A.	High income
**Turkiye**	TUR	Europe & Central Asia	1 359 123 768 774	85 518 661	1 589 272	Upper middle income
**Uganda**	UGA	Sub-Saharan Africa	53 911 907 086	50 015 092	107 791	Low income
**United Kingdom**	GBR	Europe & Central Asia	3 686 033 044 482	69 226 000	5 324 637	High income
**United States**	USA	North America	28 750 956 130 731	340 110 988	8 453 404	High income

### Overall drug availability

A total of 20 drugs or drug combinations were evaluated. Between 15 and 19 drugs were available in 21 countries (36%), 11-14 in 20 countries (34%), 5-10 drugs in nine countries (16%), 1-4 drugs in seven countries (12%), and none were available in 1 country (2%). The global distribution of drug availability is presented in Figure 1, while Figure 2 illustrates availability according to income group (Kruskal–Wallis test, *P *< .0001).

**Figure 1.**
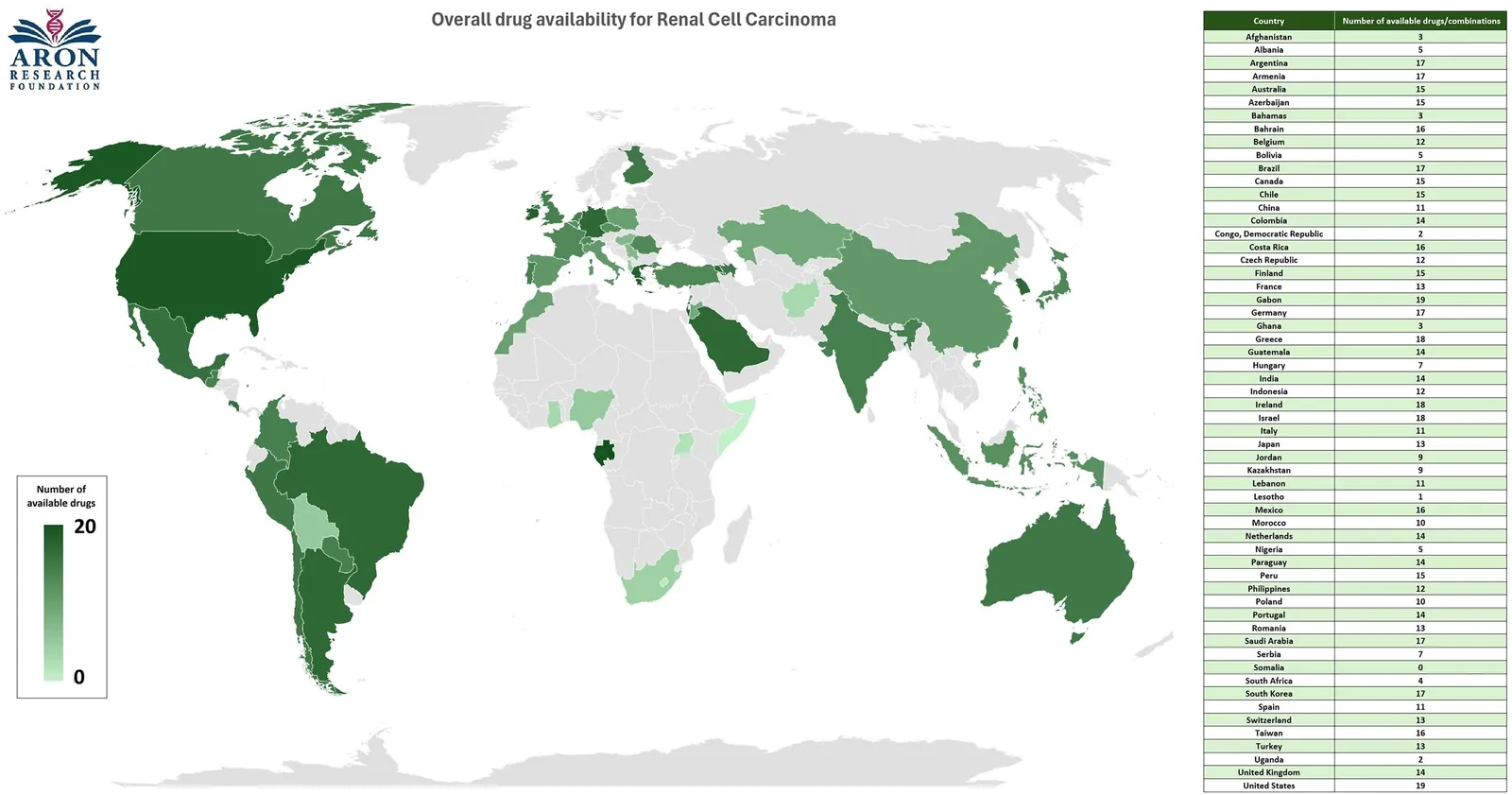
Overall geographical distribution of the selected therapies for patients with renal cell carcinoma.

**Figure 2.**
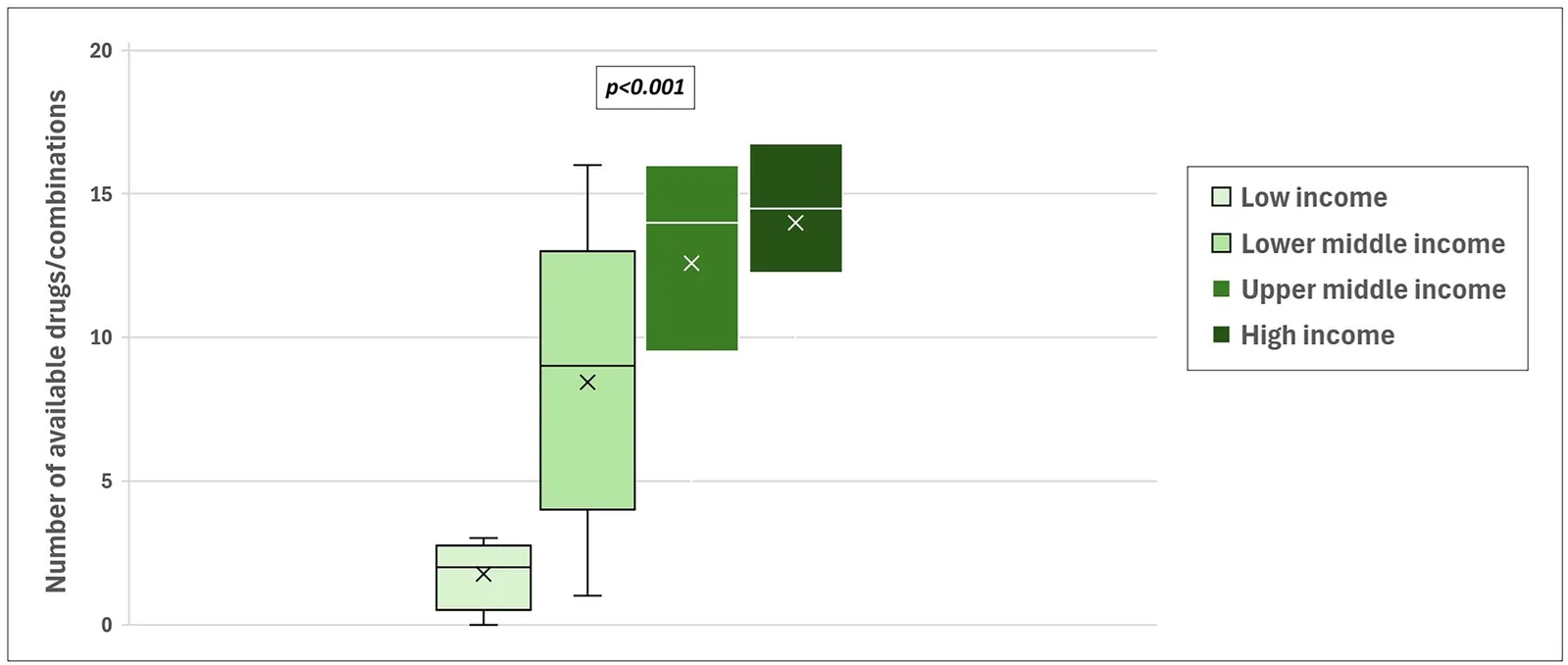
Distribution of therapies for patients with renal cell carcinoma by income group according to the World Bank Group classification.^16^ This figure presents box-and-whisker plots illustrating the distribution of the number of available therapies across country income groups. The boxes represent the interquartile range (IQR), the horizontal line within each box indicates the median, and the cross marks the mean; whiskers denote the range of the data, with outliers shown where applicable. A progressive increase in the number of available therapies is observed with rising income level, with high-income countries exhibiting the highest medians and means, as well as greater overall availability. This pattern highlights a strong positive association between national income level and access to therapeutic options.

We also examined the extent of public reimbursement for these drugs by national health systems. Full reimbursement was guaranteed by 6 countries (10%) for at least 15 of the selected innovative therapies, by 21 countries (36%) for 10-14 therapies, by 13 countries (23%) for 1-9 therapies, while in 18 countries (31%) none of these therapies was fully reimbursed (Figure 3). Detailed information on reimbursement policies for each drug or combination is provided in Supplementary Figures S3-S6.

**Figure 3.**
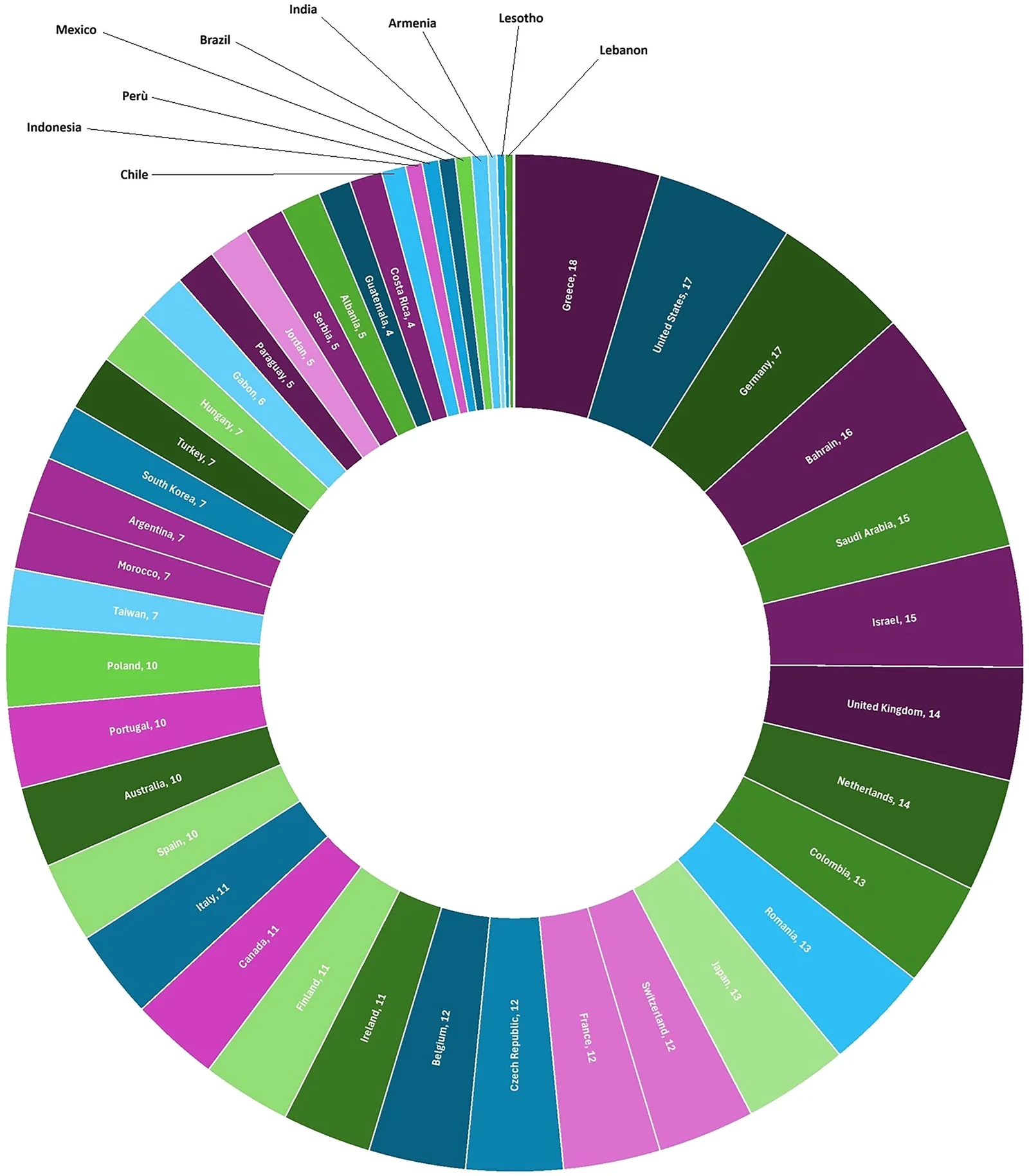
Number of drugs or combinations totally reimbursed by each public healthcare system for patients with renal cell carcinoma.

### Systemic therapies for localized disease

Two drugs were considered in the adjuvant setting: sunitinib and pembrolizumab. Sunitinib was available in 27 countries (47%), being totally reimbursed by the public healthcare system in 8 countries (14%) and only by private insurance in 16 countries (28%, Supplementary Figures S3-S6). As for adjuvant pembrolizumab, it was available in 43 countries (74%) and was totally reimbursed by the public healthcare system in 23 countries (40%) and only by private insurance in 19 countries (33%; Supplementary Figures S3-S6).

### Systemic therapies for advanced/metastatic disease

Eighteen drugs or combinations were considered in the advanced/metastatic setting. Starting from tyrosine kinase inhibitors, sunitinib the most available drug, being present in 55 countries (95%). Sunitinib was fully reimbursed by the public healthcare system in 40 countries (69%), while pazopanib in 35 (60%), sorafenib in 30 (52%), axitinib in 30 (52%), cabozantinib in 29 (50%), and tivozanib in 6 (10%; Supplementary Figures S3-S6).

As for anti-VEGF antibody bevacizumab, it was available in 41 countries (71%) and fully reimbursed in 19 (33%, Supplementary Figures S3-S6).

Concerning everolimus, it was available as monotherapy in 49 countries (84%), being fully reimbursed in 35 (60%), while the combination of everolimus and lenvatinib was available in 31 countries (53%) but only by private insurance in 15 (26%, Supplementary Figures S3-S6).

Treatment with nivolumab monotherapy was available in 44 countries (76%), whose 31 (53%) as fully reimbursed, 5 (9%) as partially reimbursed, and 8 (14%) by private insurance (Supplementary Figures S3-S6).

Among the immune-based combination therapies, nivolumab plus ipilimumab was the most available combination (42 countries, 72%), followed by nivolumab plus cabozantinib (36 countries, 62%), pembrolizumab plus axitinib (36 countries, 62%), axitinib plus avelumab (29 countries, 50%), pembrolizumab plus lenvatinib (19 countries, 33%), and axitinib pplus toripalimab (4 countries, 7%). As for drug reimbursement avelumab in 10 countries (17%), while none of analyzed countries fully reimbursed the combination of axitinib plus toripalimab, being partially reimbursed in China and Gabon and available only by private insurance in Armenia and India.

Belzutifan for patients with pretreated advanced RCC was available in 8 countries (14%) and fully reimbursed in 3 (5%), while its use in patients with von Hippel–Lindau disease was completely granted by the public healthcare system in 7 of the 15 countries (26%) in which Belzutifan is available.

### Drugs and economy

We further analyzed the relationship between countries’ 2024 GDP and the number of available drugs or combinations for RCC patients.

Armenia (0.709), Greece (0.685), and Israel (0.666) presented the higher GDP-adjusted DAI (Table 2), while India (5.195), Nigeria (4.612), and Morocco (3.794) showed the higher number of available therapies per 1000$ of GDP per capita (Figure 4). Of note, the higher number of totally reimbursed therapies per 1000$ of GDP per capita was registered in Colombia (1.643), followed by Jordan (1.089) and Lesotho (1.029; Table 2).

**Figure 4.**
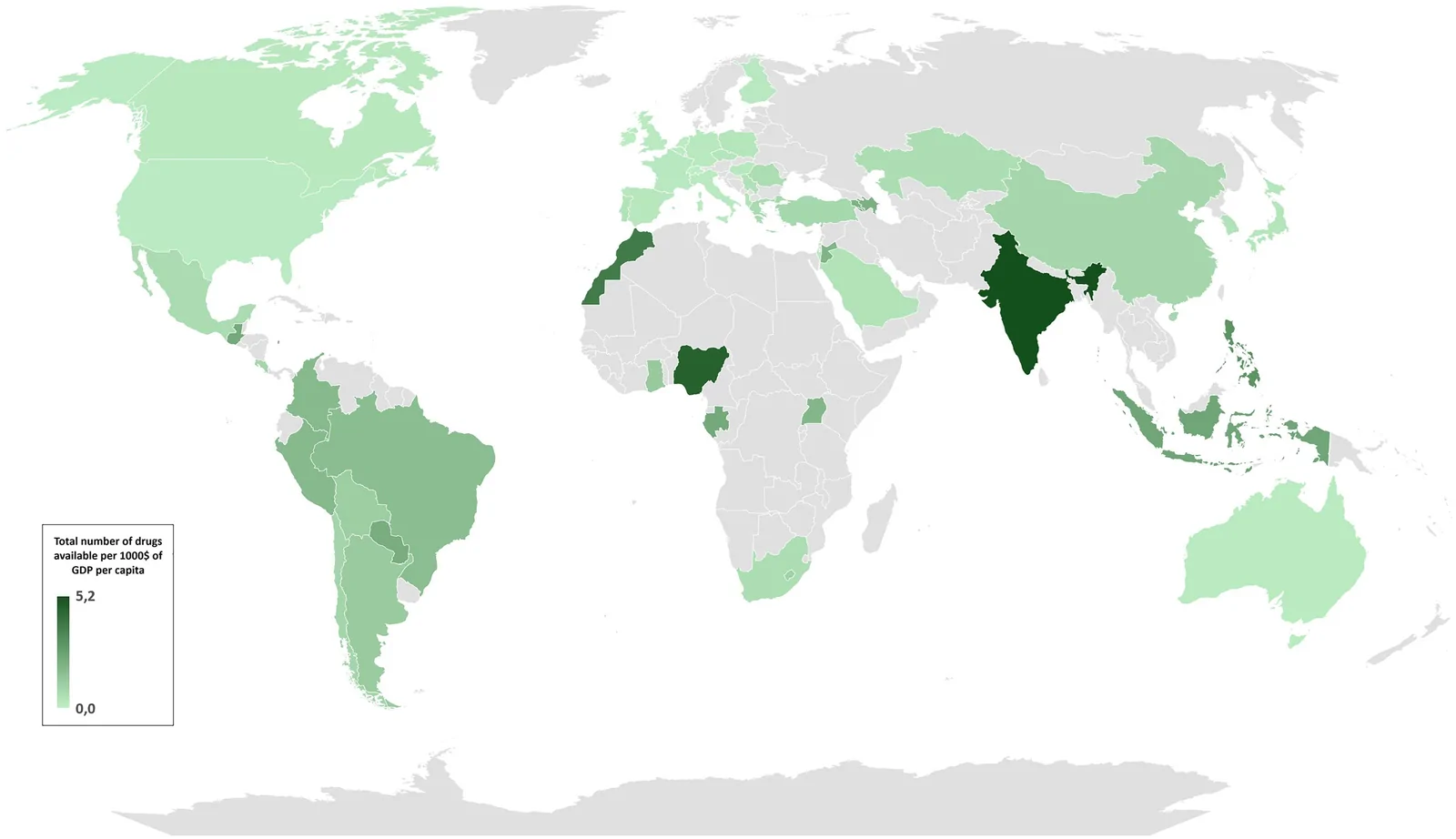
Geographical distribution of Drugs per GDP_i_.

**Table 2.** GDP-adjusted Drug Availability Index_i_ and Drugs per GDP_i_.

Country name	GDP-adjusted Drugs Availability Index	Total number of drugs available per 1000$ of GDP per capita	Number of totally reimsbursed drugs available per 1000$ of GDP per capita
**Afghanistan**	N.A.	N.A.	N.A.
**Albania**	0.208	0.439	0.439
**Argentina**	0.625	1.217	0.504
**Armenia**	0.709	1.987	0.119
**Australia**	0.532	0.232	0.155
**Azerbaijan**	0.599	2.059	0.002
**Bahamas**	0.128	0.076	0.000
**Bahrain**	0.651	0.540	0.540
**Belgium**	0.441	0.212	0.212
**Bolivia**	0.202	1.131	0.001
**Brazil**	0.598	1.649	0.195
**Canada**	0.527	0.276	0.203
**Chile**	0.566	0.898	0.180
**China**	0.360	0.827	0.008
**Colombia**	0.523	1.768	1.643
**Congo, Dem. Rep.**	0.080	3.080	0.003
**Costa Rica**	0.633	0.861	0.216
**Czechz Rep.**	0.452	0.377	0.377
**Finland**	0.568	0.282	0.207
**France**	0.452	0.282	0.261
**Gabon**	0.800	2.309	0.746
**Germany**	0.583	0.303	0.303
**Ghana**	0.119	1.255	0.001
**Greece**	0.685	0.731	0.731
**Guatemala**	0.550	2.276	0.658
**Hungary**	0.268	0.301	0.301
**India**	0.483	5.195	0.767
**Indonesia**	0.429	2.436	0.408
**Ireland**	0.663	0.159	0.098
**Israel**	0.666	0.332	0.277
**Italy**	0.386	0.272	0.272
**Japan**	0.448	0.400	0.400
**Jordan**	0.364	1.949	1.089
**Kazakhstan**	0.341	0.636	0.001
**Lebanon**	N.A.	N.A.	N.A.
**Lesotho**	0.046	1.029	1.029
**Mexico**	0.354	0.705	0.498
**Morocco**	0.620	3.794	0.485
**Netherlands**	0.503	0.207	0.207
**Nigeria**	0.190	4.612	0.005
**Paraguay**	0.571	2.182	0.793
**Peru**	0.568	1.775	0.240
**Philippines**	0.447	3.011	0.003
**Poland**	0.363	0.398	0.398
**Portugal**	0.529	0.478	0.342
**Romania**	0.487	0.647	0.647
**Saudi Arabia**	0.611	0.484	0.428
**Serbia**	0.278	0.512	0.366
**Somalia, Fed. Rep.**	0.000	0.000	0.000
**South Africa**	0.150	0.638	0.002
**South Korea**	0.602	0.469	0.193
**Spain**	0.390	0.311	0.283
**Switzerland**	0.472	0.125	0.115
**Taiwan**	N.A.	N.A.	N.A.
**Turkey**	0.465	0.818	0.441
**Uganda**	0.081	1.855	0.019
**United Kingdom**	0.484	0.263	0.263
**United States**	0.613	0.225	0.201

## Discussion

This international survey provides a comprehensive overview of global disparities in the availability and reimbursement of systemic therapies for RCC, revealing substantial inequities across countries with different income levels. Our findings demonstrate a clear gradient in drug availability and reimbursement, with high-income countries reporting significantly greater access to approved systemic therapies and combinations compared with upper–middle, lower–middle, and low–income countries. These disparities were consistently observed across geographic regions and strongly correlated with national economic indicators, supporting the hypothesis that economic capacity remains a primary determinant of access to modern oncology therapeutics.

As outlined in the Introduction, the therapeutic landscape of RCC has evolved rapidly over the past two decades, from cytokine-based regimens to targeted therapies and immune checkpoint inhibitors, culminating in the current preference for combination immunotherapy as frontline treatment.^8^ Our findings show that this therapeutic progress has not translated into equitable global access.

Despite these therapeutic advances, access to these life-prolonging therapies remains highly uneven worldwide. Our survey demonstrated that high-income countries had access to the largest number of systemic therapies, while access declined progressively across middle-income and low-income countries. These results are consistent with prior global access analyses showing that innovative oncology drugs often experience significant delays in regulatory approval and reimbursement in lower-income settings.^8^^,^^14^^,^^16^ A global study evaluating access to cancer medicines reported that many new anticancer drugs are available in high-income countries several years before becoming accessible in low- and middle-income countries, if they become available at all.^17^ This delay creates substantial disparities in treatment outcomes and contributes to global inequities in cancer survival, particularly RCC.

Economic constraints represent the most significant barrier to access and reimbursement of modern RCC therapies. Immune checkpoint inhibitors and targeted therapies are associated with high acquisition costs, which place considerable strain on healthcare systems with limited financial resources.^12^ ESMO International Consortium Study demonstrated that drug cost and national healthcare expenditure are strongly correlated with the availability of innovative therapies.^8^ Countries with higher healthcare spending per capita are more likely to provide reimbursement for novel oncology treatments, whereas countries with limited healthcare budgets often face difficult resource allocation decisions that restrict access to expensive therapies.

Reimbursement policies play a critical role in determining patient access to cancer treatments. Even when drugs receive regulatory approval, reimbursement delays and coverage limitations may prevent patients from receiving these therapies. Studies conducted in Europe have demonstrated significant variability in reimbursement timelines and coverage across countries, resulting in unequal access even within relatively high-resource regions.^18^ Similar disparities have been observed globally, with reimbursement often restricted in middle-income and low-income countries due to cost-effectiveness thresholds and budget constraints.^14^ These findings are consistent with the reimbursement disparities observed in our survey, which demonstrated substantial differences in access to fully reimbursed therapies across income groups.

Notably, no single country had access to all 20 evaluated therapies, and conversely, no individual drug was available across all 58 participating countries. This finding indicates that disparities in RCC drug access are not confined to low-resource settings alone, but reflect a broader, fragmented global landscape in which even high-income countries lack full access to the complete portfolio of guideline-recommended options, likely reflecting country-specific regulatory timelines, market-entry decisions by individual manufacturers, and national formulary priorities, rather than income disparities alone.

In addition to economic barriers, structural and regulatory factors contribute to disparities in drug availability. Differences in national regulatory frameworks, healthcare infrastructure, and pharmaceutical market dynamics influence the timing and extent of drug introduction.

The disparities identified in this study have important clinical implications. Limited access to effective therapies may result in inferior outcomes for patients with RCC in resource-constrained settings. Population-based studies have demonstrated that cancer survival rates are significantly lower in low- and middle-income countries compared with high-income countries, reflecting differences in both diagnosis and treatment availability.^19^ Ensuring equitable access to effective systemic therapies is therefore essential to improving global cancer outcomes.

Strategies such as differential pricing, expansion of reimbursement programs, increased participation in clinical trials, and improved regulatory harmonization may help reduce disparities in drug access. Additionally, the development of biosimilars and generic therapies may improve affordability and accessibility over time. Actual patient-level access, which may be influenced by reimbursement restrictions, institutional policies, and regional healthcare infrastructure. Additionally, affordability and out-of-pocket costs were not directly evaluated.

For settings where the full spectrum of first-line combination regimens is not readily accessible, several pragmatic, guideline-concordant strategies may help optimize outcomes within existing resource constraints. Adjuvant therapy should be prioritized where potentially curative, as a subset of patients with resected localized or locally advanced disease may achieve durable remission. In the metastatic setting, single-agent TKI therapy (eg, sunitinib or pazopanib), which were considerably more available and reimbursed in our survey than combination regimens, may represent a reasonable option for patients with IMDC favorable-risk disease when combination immunotherapy is not accessible, consistent with current guideline recognition of risk-adapted treatment approaches.^9^ More broadly, tiered/differential pricing agreements, expanded access and compassionate-use programs, and regional pooled-procurement mechanisms may help narrow the gap in resource-constrained health systems.

In conclusion, this international survey demonstrates significant global disparities in the availability and reimbursement of systemic therapies for RCC, strongly associated with national income level and healthcare expenditure. While therapeutic advances have dramatically improved RCC outcomes, these benefits remain inequitably distributed worldwide. Addressing disparities in drug access and reimbursement represents a critical step toward achieving global equity in cancer care and ensuring that therapeutic innovation translates into improved outcomes for patients across all regions.

## Supplementary Material

oyag397_Supplementary_Data

## Data Availability

Data are available upon reasonable request and subjecttohical/legal restrictions.
